# New Insights in Early Detection of Anticancer Drug-Related Cardiotoxicity Using Perfusion and Metabolic Imaging

**DOI:** 10.3389/fcvm.2022.813883

**Published:** 2022-02-07

**Authors:** Farah Cadour, Franck Thuny, Joevin Sourdon

**Affiliations:** ^1^Aix-Marseille Université, CNRS, CRMBM, Marseille, France; ^2^APHM, Hôpital Universitaire Timone, CEMEREM, Marseille, France; ^3^Aix-Marseille University, University Mediterranean Center of Cardio-Oncology, Unit of Heart Failure and Valvular Heart Diseases, Department of Cardiology, North Hospital, Assistance Publique - Hôpitaux de Marseille, Centre for CardioVascular and Nutrition Research (C2VN), Inserm 1263, Inrae 1260, Marseille, France

**Keywords:** cardio-oncology, cardiotoxicity, perfusion, metabolism, mitochondria, magnetic resonance spectroscopy or MRS, magnetic resonance imaging, nuclear imaging

## Abstract

Cardio-oncology requires a good knowledge of the cardiotoxicity of anticancer drugs, their mechanisms, and their diagnosis for better management. Anthracyclines, anti-vascular endothelial growth factor (VEGF), alkylating agents, antimetabolites, anti-human epidermal growth factor receptor (HER), and receptor tyrosine kinase inhibitors (RTKi) are therapeutics whose cardiotoxicity involves several mechanisms at the cellular and subcellular levels. Current guidelines for anticancer drugs cardiotoxicity are essentially based on monitoring left ventricle ejection fraction (LVEF). However, knowledge of microvascular and metabolic dysfunction allows for better imaging assessment before overt LVEF impairment. Early detection of anticancer drug-related cardiotoxicity would therefore advance the prevention and patient care. In this review, we provide a comprehensive overview of the cardiotoxic effects of anticancer drugs and describe myocardial perfusion, metabolic, and mitochondrial function imaging approaches to detect them before over LVEF impairment.

## Introduction

Cancer therapy significantly improves patient survival but is sometimes accompanied by cardiotoxic effects. Cardiotoxic complications can range from myocardial abnormalities, valvular abnormalities, pericardial diseases, coronary artery disease (CAD), and alteration in left ventricle ejection fraction (LVEF).

Anthracyclines, one of the most used and oldest chemotherapies, are the archetypal cardiotoxic anticancer drug, ultimately leading to the heart failure (1). In addition, the emerging field of cardio-oncology has seen the development of new anticancer drugs such as antiangiogenics also leading to cardiotoxicity with endothelial dysfunction, forcing a reconsideration of the stages, timing, and levels of cardiotoxicity.

Initial evaluation of LVEF and subsequent evaluation under anticancer therapy is paramount as the most guidelines for cardiotoxicity are based on LVEF impairment (2). To date, echocardiography remains the most frequently used method to detect LVEF alteration, but also by assessment of left ventricle (LV) longitudinal strain evaluation that might identify early LVEF dysfunction (3). Although not considered the first-line method, cardiac magnetic resonance imaging (CMR) can assess cardiac anatomy, structure, and tissue properties in addition to LVEF.

These modalities have been able to detect impaired cardiac function in the later stages of cardiac side effects (4). Myocardial perfusion imaging and metabolic imaging are powerful approaches providing novel biomarkers that can improve early detection of cardiotoxicity before irreversible cardiac damage occurs. This review summarizes the alterations in cardiac perfusion and metabolism that occur in anticancer drug-related cardiotoxicity and the advantage of assessing perfusion and metabolism non-invasively in the beating heart with cardiac imaging.

## Myocardial Vascular and Metabolic Effects of Anticancer Drugs

### Overview of the Link Between Myocardial Circulation and Metabolism

There is a close relationship between myocardial blood circulation, which delivers oxygen and nutrients, tissue metabolism, and oxidative stress. The heart has a very high energy demand to sustain contractile function and synthesizes adenosine triphosphate (ATP) through oxidative metabolism of free fatty acids (FFA), glucose, ketones, and lactate (5).

The adult heart normally obtains 50–70% of its ATP from fatty acid β-oxidation in the presence of oxygen. However, it must adapt, switching from one substrate to another, to sustain demand depending upon the metabolic state and physical conditions at the time (5). Under well-perfused aerobic conditions, glucose and FFA are catabolized into pyruvate or acyl-CoA, respectively, both of which are catabolized to acetyl-CoA to enter the tricarboxylic acid (TCA, Krebs) cycle. Most of the energy supply is then derived from the mitochondrial oxidative phosphorylation system. The main cardiac energy reserve is phosphocreatine (PCr), which is maintained by the following creatine kinase (CK) reaction:


$$\begin{array}{l} PCr+ADP+H^{+}↔ATP+Creatine \end{array}$$

This system facilitates intracellular delivery of energy from mitochondria to cytoplasmic sites of ATP utilization and maintains a high level of ATP during changes in energy demand (6).

Direct damage to the mitochondria, blood supply, and myocardial metabolism will be responsible for abnormal production of reactive oxygen species (ROSs). ROS are reactive intermediates of the molecular oxygen that are essentially generated during mitochondrial oxidative phosphorylation (7). Cellular sources of ROSs are cardiomyocytes, endothelial cells, stromal cells, and inflammatory cells in the heart (8). One of the major ROSs is the proximal mitochondrial ROSs (superoxide anion), which can be generated by a loss of ATP production or when there is a high NADH/NAD^+^ ratio in the mitochondrial matrix (9). An imbalance between ROS production and antioxidant cell response leads to endothelial dysfunction, the release of proinflammatory cytokines, and vasoconstriction of epicardial and microvascular coronary arteries (10). The heart is particularly sensitive to oxidative stress because of its low-antioxidant resources (11–13). One of the main mechanisms of ROS leading to endothelial dysfunction is the uncoupling of endothelial nitric oxide (NO) synthase, which usually facilitates NO production (14), ultimately leading to reduce NO bioavailability. Indeed, the endothelium synthetizes the NO (15), which acts as a vasodilator, an antithrombotic, and an anti-atherosclerotic molecule (14). Endothelial nitric oxide synthase (eNOS) is the type III of NO synthases (NOS) that will lead to NO radicals synthesis from L-arginine and is expressed in endothelial cells. But in the inflammatory situation, the other NO synthases are neuronal NOS (type I) and inducible NOS (iNOS, type II). The latter will be expressed in blood vessels under pathological conditions such as inflammation or oxidative stress (16). Major cell structure and function damages will result reaction of NO with superoxide anion leading to peroxynitrite (17).

Interestingly, initial vascular injury also results in the production of ROSs species derived from NAD(P)H (18). Oxidative inflammation will ultimately cause adventitial fibrosis and smooth muscle hypertrophy (18). The latter phenomenon can also be observed in the media and intima through paracrine effects of adventitial inflammation. As a result, medial layers of vessels do not respond to NO to adapt blood flow and assure normal myocardial perfusion (19), resulting in impaired endothelium-dependent relaxation.

It is important to bear in mind that impaired myocardial perfusion and/or subsequent alteration of metabolic pathways, substrate preferences, and bioenergetics (i.e., reduced PCr/ATP ratio) might contribute to the development of several common cardiovascular diseases (20). For these reasons, perfusion and metabolic imaging are preferred methods to study early vascular and metabolic cardiotoxic effects.

### Anticancer Drugs

The vascular and metabolic cardiotoxic effects of the various anticancer drugs are given in Table 1.

**Table 1:** Myocardial vascular and metabolic effects of common anticancer drugs.

**Anticancer drugs**	**Mechanisms of cardiotoxicity**
Anthracyclines	Microcirculation alteration
	Endothelial dysfunction (NO)
	Microcirculation increased thickening
	Altered oxidative metabolism
	Impaired energetics
	ROS
	Mitochondrial dysfunction
Antimetabolites	Vasospasm
	Vasoconstriction
	Endothelial dysfunction (NO)
	Smooth cell dysfunction
	Altered oxidative metabolism
	Impaired energetics
	Mitochondrial dysfunction
	ROS
RTKi	Inhibits angiogenesis
	Endothelial dysfunction (NO)
	Vasoconstriction
	Altered oxidative metabolism
	Myocardial insulin resistance pattern
	Impaired energetics
	ROS
	Mitochondrial dysfunction
Anti-VEGF Ab	Inhibits angiogenesis
	Capillary rarefaction
	Impaired energetics
	ROS
	Mitochondrial dysfunction
Anti-HER2 Ab	Microcirculation alteration (neuregulin 1)
	Disruption of cardioprotective *Neuregulin-1* pathway
	ROS
	Mitochondrial dysfunction
ICI	Microcirculation alteration → vascular sequelae
	Dysregulated myocardial metabolism
Taxanes	Impaired energetics
	Endothelial damage
	Capillary rarefaction
Alkylating agents	Endothelial dysfunction (NO)
	ROS
	Free fatty acids accumulation
	Vasoconstriction
	Mitochondrial dysfunction

*Ab, antibody; NO, nitric oxide; ROS, reactive oxygen species*.

#### Anthracyclines

Anthracyclines are a group of chemotherapy broadly used in cancer treatment, with doxorubicin (DOX) being one of the most widely used. Its cardiotoxicity is well-known with cumulative toxicity ultimately leading to permanent cardiac alteration (21). The initial alteration of this end state is thought to be at a microvascular level through ROS production (22–24), with mitochondrial superoxide production increasing with DOX dose (25).

Excessive production of ROS by DOX leads to apoptosis, cardiac function impairment, inflammation, and vascular injury (25, 26). Both the cardiomyocytes and arterial endothelial cells can experience mitochondrial dysfunction under anthracyclines (27, 28). These properties suggest that, in addition to its known direct effect on deoxyribonucleic acid through topoisomerase II beta inhibition (29), endothelial cells injury could be one cause of anthracycline cardiotoxicity. Although anthracyclines cardiotoxicity is usually detected at a stage of altered ejection (21), studies suggest that anthracyclines cardiotoxicity occurs in a continuum, challenging the hypothesis of irreversible cardiac injury (30, 31).

Current guidelines suggest monitoring of patients with cancer undergoing chemotherapy by echocardiography since most definitions of cardiotoxicity are based on LVEF decline (2), but the literature reports microcirculation changes long before any LVEF or contraction alterations occur (31, 32). This myocardial perfusion alteration could be the result of increased arterial walls thickening, which can occur early and even after a single DOX injection (31, 33), but is more overt with repeated injections (33). The increase in intima-media thickness under anthracyclines (34) is in part secondary to oxidative inflammation. Thus, anthracyclines cardiotoxicity appears at the histological level and these microcirculation alterations appear to be an early form of the well-known anthracyclines cardiotoxicity, suggesting modalities to assess the initial endothelial cell damage and better prevent its progression. Moreover, the combination of radiotherapy with anthracyclines potentiates heart damage. Radiotherapy has been reported as responsible for cardiac perfusion defect development, however, myocardial perfusion imaging of the combination of radiotherapy with anthracyclines remains poorly described (32).

#### Antimetabolites

5-Fluorouracil (5-FU) is a part of antimetabolite agents and is commonly used in the treatment of malignancies. One of the major cardiotoxicities of 5-FU is coronary vasospasm that can lead to ischemia. Its mechanism remains uncertain, with some suggesting an endothelial-dependent mechanism through endothelial dysfunction, but others an endothelium-independent with vasoconstriction of dysfunctional smooth muscle cells (35). Studies in animal models demonstrated that altered erythrocyte metabolism decreases erythrocyte ability to bring oxygen to the myocardium (36, 37). 5-FU reduces oxidative metabolism (38), impairs energetics (38), and induces mitochondrial uncoupling reducing aerobic efficiency (39). At a subcellular level, the toxicity of 5-FU and another antimetabolite drug, the capecitabine, have been shown to be mediated through oxidative stress with ROS generation leading to altered mitochondrial membrane potential in isolated rat cardiomyocytes (40).

#### Alkylating Agents

One of the main alkylating agents, mostly used in hematologic cancers, is cyclophosphamide, for which dose-mediated cardiotoxicity is one of the notable toxic effects. The metabolites of cyclophosphamide reported to be involved in cardiotoxicity are acrolein and 4-hydroxy-cyclophosphamide. These metabolites are involved in ROS generation (41, 42) that damage mitochondrial membrane by decreasing its detoxifying capacity, but also by disrupting normal vasotone response pathway through NO reduction or an increase in the vasoconstrictor endothelin-1 (23). In addition, cyclophosphamide is responsible for FFA accumulation and reduction of ATP production resulting in the release of proinflammatory cytokines (41). Cardiac microscopic findings of alkylating agents consist of interstitial damages, myocardial necrosis, vacuolar changes, and intramural changes in small coronary vessels (43). Similar disturbances have also been reported with cisplatin-based chemotherapy, another alkylating agent (44).

#### Taxanes

Taxanes are antimicrotubules whose main cardiotoxicity is disruption of cardiac rhythm and conduction. Heart failure (possibly in combination with DOX), ischemia, and microvascular rarefaction because of the endothelial damage might also occur (45).

#### Receptor Tyrosine Kinase Inhibitors

Receptor tyrosine kinase inhibitors (RTKi) include sorafenib, pazopanib, and sunitinib. As a part of antiangiogenic therapy, RTKi inhibits the tyrosine kinase activity of the vascular endothelial growth factor (VEGF) receptor, thereby blocking the VEGF pathway, but also platelet-derived growth factor receptors and c-kit (46). Oxidative stress and dysregulation of NO signaling have been proposed to mediate RTKi-induced hypertension, as they are known to be involved in the VEGF pathway (47, 48). However, sunitinib-induced hypertension has been associated with upregulation of the endothelin peptide (49–51), a potent vasconstrictor known to induce cardiac endothelial dysfunction (52). Experimental studies investigating the effects of VEGFR blockade on cardiac microvasculature did not reveal any changes in the number of capillaries (50, 53). Nevertheless, sunitinib induces a loss of coronary microvascular pericytes in mice (53), which might explain the impaired coronary flow reserve (CFR) of sunitnib-induced cardiotoxicity (49, 53).

Carbohydrate metabolism is altered in the myocardium of sunitinib-treated mice, which exhibits higher glucose uptake, higher gene expression of pyruvate dehydrogenase kinase, and of the pyruvate kinase isoform 2 (54), a signature of fetal myocardium in which the metabolism is mostly anaerobic. The sensor of cardiac energetic metabolism, AMP-activated protein kinase, is inhibited by sunitinib (55). Energy impairment because of the loss of mitochondrial membrane potential resulting in reduced ATP has been reported in the early stages of sunitinib-treated cardiomyocytes (56).

In a comparative study, only sorafenib among others RTKi directly impaired mitochondrial function and oxidative metabolism at clinically concentrations (57), but ROS generation was documented in several RTKi-treated myocardium (58, 59).

#### Anti-vascular Endothelial Growth Factor (VEGF) Monoclonal Antibody

Another antiangiogenic approach consists of blocking VEGF with a humanized monoclonal antibody, which traps endogenous VEGF and inhibits its binding with the receptor. Bevacizumab was the first anti-VEGF antibody with a rate of sytemic hypertension as high as 70%, probably because of the vascular resistance, endothelial dysfunction, and capillary rarefection (39). Bevacizumab induces mitochondrial dysfunction plus ROS formation in isolated rat heart (60, 61) and in isolated cardiomyocytes (62).

#### Anti-human Epidermal Growth Factor Receptor (HER 2)

Human epidermal growth factor receptor 2 is a receptor that promotes cell growth, proliferation, and repair in the body. Tumors can hijack these functions to proliferate. Therefore, one treatment option is to specifically target this receptor, with anti-HER2 therapy, led by Trastuzumab, which has revolutionized the treatment and prognostic of patients with HER2 positive breast cancer (63). Trastuzumab will result in ROS production, mitochondrial dysfunction, and proapoptotic signals release in cardiomyocytes (64). Unlike anthracyclines, cardiotoxicity of anti-HER2 is dose-independent and often reversible. However, it results in greater cardiotoxicity in the presence of or after anthracyclines (65).

Anti-HER2 might cause cardiomyocyte damage by disrupting the neuregulin-1 axis that normally activates protective pathways in response to stress (66), which could lead to LVEF decrease. Neuregulin-1 is a cardioactive growth factor that normally participates in the dimerization of HER receptors on cardiomyocytes to provide cell protection. However, the fact that neuregulin-1 is released from the endothelial cells in the heart leads to the question of whether the impaired LVEF is due to a direct impact of anti-HER2 on cardiomyocytes or an indirect impact *via* endothelial cells of the altered coronary microvasculature (67). Interestingly, a decrease in neuregulin-1 levels has been associated with CAD (68). The same neuregulin-1/HER pathway may also explain the increased susceptibility to anthracyclines cardiotoxicity when the two treatments are combined.

#### Immune Checkpoint Inhibitors (ICIs)

Immune checkpoint inhibitors are monoclonal antibodies that restore antitumor immunity by targeting inhibitory receptors on the lymphocytes surface, such as cytotoxic T-lymphocyte-associated protein 4, programmed cell death receptor 1 (PD1), and its ligand. By reactivating the immune response against the tumor, ICIs can lead to immune-related cardiovascular adverse events that, although rare, present a case-fatality rate as high as 50% (69). The most-reported cardiac complications of ICIs are ICI-induced myocarditis but also pericardial diseases, cardiomyopathy, myocardial fibrosis, and acute heart failure (70). Microvascular damage leading to vascular sequelae has also been reported with ICI (10). Furthermore, studies are needed to explore all the different pathways involved in the cardiotoxicity of ICIs with possible yet unknown microcirculation damage. A recent *in vivo* study in mice showed that anti-PD1 drugs cause myocardial dysfunction and altered myocardial metabolism, suggesting damage at a subcellular level (71).

## Imaging

**Figure 1:**
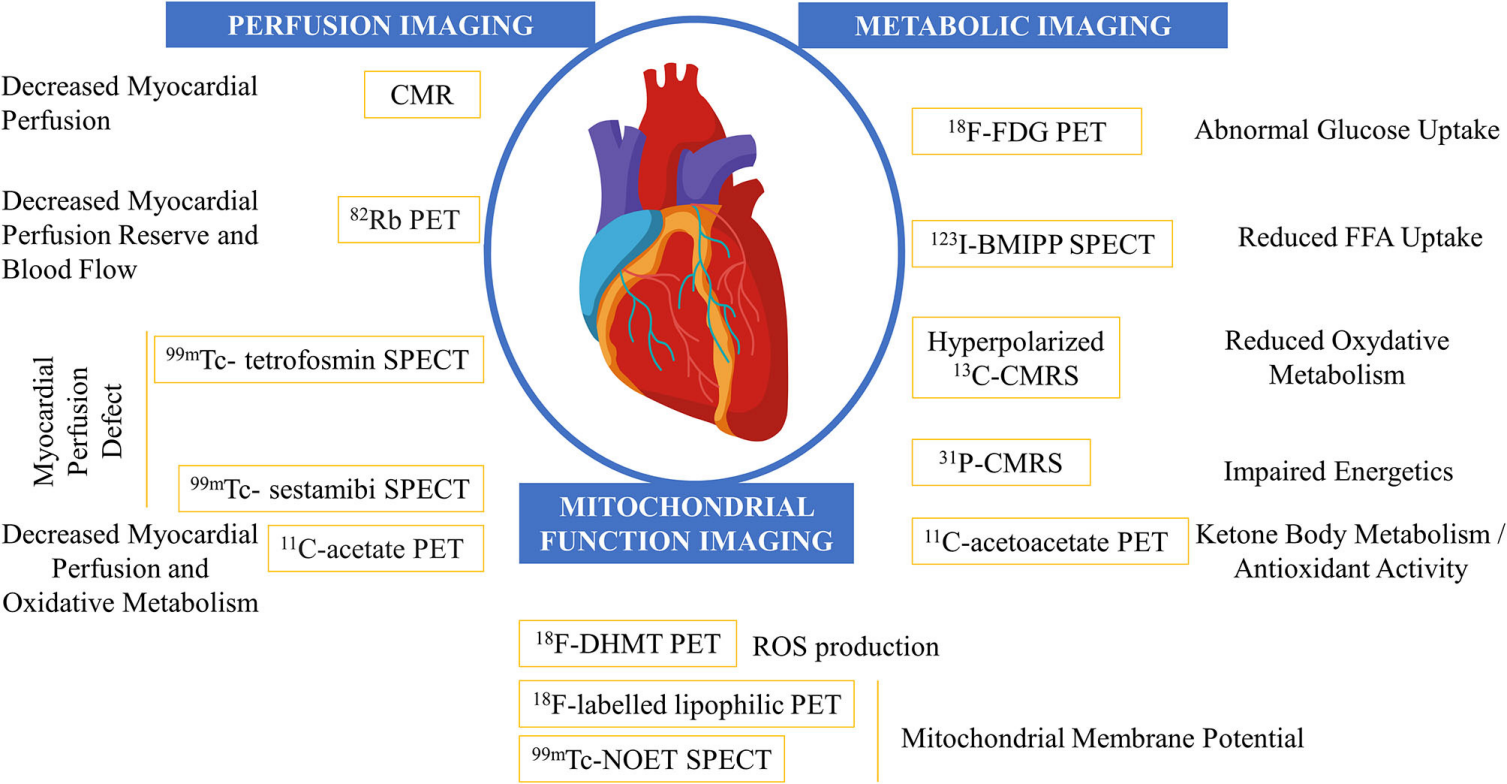
Imaging modalities in the field of cardio-oncology and their assessment of anticancer-drug related cardiotoxicity. CMR, cardiac MRI; CMRS, cardiac magnetic resonance sprectroscopy; FDG, fluoro-D-glucose; FFA, free fatty acid; SPECT, single-photon emission CT.

Imaging modalities in cardio-oncology and their assessment of anticancer-drug-related cardiotoxicity are given in Figure 1.

### Perfusion Imaging

Perfusion imaging involves assessing the delivery of oxygen and nutrients to tissues through blood flow. It aims to describe microvasculature that can be altered under the effect of anticancer drugs. Since 1997, Hasdai et al. reported that coronary endothelial dysfunction may be associated with myocardial perfusion defects (72). Both radiotherapy and chemotherapy have shown to be associated with microvascular dysfunction (2), although the effect of non-radiation therapies on the latter is less well-described (31). Knowing the effects of anticancer drugs on myocardial microcirculation, myocardial perfusion imaging appears to be an attractive modality to detect anticancer drug-related myocardial toxicity. Moreover, by the time cardiotoxicity-associated LV dysfunction is detectable by echocardiography, it is often too late, emphasizing the need to assess the initial microvasculature dysfunction to better prevent it.

Symptomatic oxygen supply-demand mismatch can be evaluated invasively by invasive coronary angiography (ICA), but myocardial microcirculation disturbance can occur before any visible epicardial coronary on ICA (73), requiring blood flow measurements to assess myocardial function. Myocardial malperfusion can be unmasked through fractional flow reserve (FFR), which is an invasive measurement under hyperemia to determine the significance of an epicardial coronary artery stenosis, with an FFR ≤ 0.80 considered to be ischemia prone (74), and defined as the ratio of maximal blood flow distal to proximal to the stenosis. The invasive measurement of CFR is intended to study the vascular bed and describe the myocardial reserve capacity for vasodilatation, and is defined as the ratio of maximal hyperemic to the resting coronary blood flow (75).

Another interesting measure to evaluate coronary microvascular dysfunction is the index of microcirculatory resistance (IMR) (76) which is an index of coronary microvasculature and considered as abnormal if ≥25 independently of epicardial stenosis (77). However, these different parameters remain invasive, which could explain their low use in clinical practice for monitoring patients undergoing anticancer therapy, and should be discussed with respect to non-invasive techniques for the assessment of myocardial perfusion, which we review here.

#### Nuclear Imaging

Nuclear imaging techniques include single-photon emission computerized tomography (SPECT) and PET. These techniques are based on the detection of radioactive gamma rays and photons (after positrons annihilation) from an injected radioactive compound, respectively.

##### Single-Photon Emission CT (SPECT)

Impairment of epicardial arteries vasodilatation, by evaluation of change in coronary diameters under pharmacological stress, has been reported after DOX infusions on CT angiography suggesting dysfunction of smooth cells and the microvascular bed (78). However, the resolution of cardiac CT is insufficient to visually assess microvessels, underlining the need for cardiac perfusion CT to assess myocardial microcirculation by detecting hypoperfused territories. Coupling of metabolic information by traditional radiotracers ^201^Tl-chloride, ^99m^Tc- sestamibi, and ^99m^Tc- tetrofosmin, is obtained by myocardial perfusion SPECT. SPECT is performed at rest and under stress, which can be achieved by exercise or pharmacologically with vasodilators (79). The added value of SPECT is that the radiotracers will be delivered to the myocardium in proportion to flow and therefore be able to unmask a myocardial perfusion defect secondary to CAD. Territories with myocardial perfusion abnormalities may not only be secondary to CAD but reflect the myocardial cardiotoxicity at a microvascular level.

Studies have reported increased perfusion defects in DOX-treated patients with a history of radiotherapy (32, 80). Galluci et al., have suggested myocardial perfusion abnormalities, assessed by SPECT, without LVEF dysfunction in patients who had undergone chemotherapy and radiotherapy (32). However, this observational study could not strictly conclude that the findings were only due to the chemotherapy because of the lack of a control group before cancer treatment, and because of the inclusion of patients with a history of radiotherapy.

Some studies described LVEF dysfunction after the introduction of DOX in patients with cancer (81), but there are very little data on the incidence of SPECT perfusion defect in patients under DOX alone. One study on 36 patients with breast carcinoma evaluated before and after anthracyclines found no significant perfusion defect after anthracyclines (34), leaving the question of myocardial perfusion monitoring with ^99m^Tc-sestamibi SPECT subject open to debate.

##### Positron Emission Tomography

Compared with SPECT, PET allows assessment of myocardial blood flow with better spatial resolution and sensitivity. CFR can be quantified as the ratio of myocardial blood flow between stress and rest on PET (82). The most commonly used and validated radionuclide for cardiac perfusion evaluation is rubidium-82 (^82^Rb) (82). Although ^82^Rb PET is often used for semiquantitative myocardial perfusion, it may assess coronary microvascular function by absolute quantification of myocardial perfusion and myocardial perfusion (or flow) reserve (MPR) (83). MPR is the ratio of stress flow to resting flow and describes the capacity of the coronary bed to maximize flow (84).

Myocardial perfusion reserve has been reported to be decreased after DOX exposure, representing a possible early marker of DOX myocardial cardiotoxicity (85). Detection of changes in mitochondrial function, estimation of myocardial blood flow and myocardial oxygen consumption, and thus, the ability of coronary arteries to respond to stress, can also be assessed by ^11^C-acetate rest stress PET. Using the latter, a decrease in myocardial perfusion and oxygen consumption reserve in DOX-treated rats compared with the control animals has been reported (86). ^11^C-acetate PET is not only used to investigate DOX cardiotoxicity but has also been evaluated in sunitinib-induced cardiotoxicity. Similarly, an *in vivo* study in rats described a decrease in myocardial perfusion, evaluated by ^11^C-acetate PET, as early as 5 days after treatment initiation (87).

#### Cardiac MR

Common practices remain the assessment of cardiotoxicity by echocardiography because of its ability and availability to detect LVEF alteration, which is the current standard for oncologic treatment cardiotoxicity (88). However, the gold standard in LVEF evaluation remains CMR imaging (89). But in addition to LVEF assessment, it is currently admitted that CMR with vasodilator stress perfusion should be performed to non-invasively investigate microvascular dysfunction (90). Yet, we know that anthracyclines may be responsible for myocardial damage at a histologic level long before any overt LVEF decrease (91). Although most studies of anthracyclines have focused on their effect on myocyte damages (92), more recent studies suggest that DOX cardiotoxicity may present as direct vascular injury and arterial damage with coronary arteriolar wall abnormalities (31, 33, 93, 94). Some mechanisms of microcirculation damage arise from increased thickening of microcirculatory arterioles and loss of smooth muscle cells, which may contribute to myocardial perfusion defects.

Thus, the literature reports that DOX cardiotoxicity results in microvascular dysfunction, and we know that microvascular can technically be assessed by myocardial perfusion on CMR. We had to wait until 2021 to finally find a study that proved *in vivo* that there was a reduction in myocardial perfusion well before any overt LVEF alteration. Indeed, to the best of our knowledge, Galán-Arriola et al. (31) were the first to describe in large animals the impact of DOX on coronary microcirculation, assessed by CMR but also by invasive measurement and histology, under different DOX protocols. In this study, the alteration of myocardial perfusion by CMR followed a similar pattern to that observed in the assessment of microcirculatory function by CFR. Indeed, they showed that in the early stages of DOX treatment, there was a decline in CMR perfusion. This decline in perfusion was present although LVEF, cardiac motion, cardiac contractility were not impaired; and was persistent as long-term changes with cumulative doses of DOX.

Myocardial perfusion assessment by CMR is a validated non-invasive assessment of microvascular CAD (95) and has been shown to outperform SPECT in detecting obstructive CAD (96–99). Newer CMR techniques that could quantitatively detect epicardial and microvascular CAD have correlated well with IMR and FFR measurements (77), and coronary sinus flow evaluation could be a good surrogate for CFR measurements (100). Although to the best of our knowledge, no study has yet reported myocardial perfusion CMR findings of anthracyclines-treated patients, it is legitimate to speculate that vasoconstriction and increased wall thickness of the heart microvasculature may reveal a myocardial perfusion defect and decreased myocardial blood flow reserve. Myocardial perfusion is acquired during the first pass of gadolinium-based contrast agents, based on an ECG-triggered fast T1-sensitive pulse sequences that can be acquired both at rest and with stress. The additional benefit of stress in CMR perfusion compared with resting perfusion alone is still debated but is theoretically used to unmask myocardial perfusion defect that could be compensated at rest (101). Indeed, stress could reveal insufficient coronary reserve resulting in decreased perfusion and ischemia in territories with thickened vessels walls and impaired ability to respond to stress-induced vasodilation. Although the mechanisms leading to 5-FU-related cardiotoxicity are numerous and detailed elsewhere (102), ischemia, especially secondary to vasospasm, can be imaged by perfusion defect in the coronary territory of the vasospasm (103, 104).

Regarding the evaluation of anti-VEGF myocardial cardiotoxicity with perfusion CMR, there are very sparse data in the literature. A small study on 9 patients evaluated both resting and stress perfusion with CMR before treatment and at 4 and 6 weeks of treatment (105). They were able to show a decrease in myocardial blood flow on resting perfusion after treatment introduction but no difference under stress, and an increase in vascular permeability. These preliminary findings suggest that anti-VEGF cardiotoxicity leads to microvascular constriction, which may, fortunately, be reversible, and that microvascular endothelial dysfunction may be responsible in part for impaired LVEF.

### Metabolic Imaging

Metabolic imaging focuses and targets changes in metabolic pathways and energetics. It includes CMRS and nuclear imaging techniques such as SPECT and PET.

#### Cardiac Magnetic Resonance Spectroscopy

Cardiac magnetic resonance spectroscopy has several advantages for metabolic imaging since it is able of measuring several metabolic biomarkers without using ionizing radiation (106). Metabolites containing proton (^1^H) such as creatine or lipids; containing carbon (^13^C) such as glucose, and containing phosphorus (^31^P) such as PCr or ATP can be assessed by CMRS. In addition, the development of ^31^P saturation magnetic resonance spectroscopy allows the measurement of the metabolic rate of ATP production *via* the enzyme creatine kinase (= CK flux) (106, 107).

Early studies performed on isolated animal hearts have demonstrated several alterations in the cardiac metabolic. The injection of [1-^13^C]glucose into isolated perfused hearts treated for 10 weeks with anthracyclines highlighted altered glycolytic metabolism (108). Similarly, abnormal cardiac bioenergetics, as revealed by a reduced PCr/ATP ratio, was measured with ^31^P-CMRS in an isolated animal hearts of acute (109) and chronic (110–112) anthracycline-related cardiotoxicity. In addition, Bittner et al. showed that hearts chronically exposed to DOX failed to adapt metabolically, as evidenced by the delayed recovery of PCr after hemodynamic stress (113). Recently, Henderson et al. showed that acute and clinically relevant exposure to DOX in isolated, perfused rat hearts induced a reduction in energy reserve, as measured by a decrease in PCr, in response to the cardiac-stimulant isoproterenol (114). These studies demonstrated abnormal cardiac energetics production and utilization, even in the setting of acute anthracycline exposure. Interestingly, the myocardial PCr/ATP ratio was reduced after 6 weeks of anthracycline treatment without evidence of cardiac damage in an *in vivo* study (110). In addition, the authors showed a strong correlation between cardiac energetics and LV systolic and diastolic dysfunction after 8 and 10 weeks of treatment. The same group then demonstrated that the absolute concentration of PCr was decreased in DOX-treated mice and that ^31^P-CMRS also detected a reduced rate of ATP synthesis through CK reaction (115). Importantly, overexpression of cardiac-specific myofibrillar isoform of CK restored impaired PCr and CK flux, which was associated with improved LVEF and survival in DOX-treated mice (115), opening up a new possibility for preventive therapy.

Recent research has focused on improving the signal-to-noise ratio of conventional CMRS, with the development of hyperpolarization CMRS: the injection of hyperpolarized [1-^13^C]pyruvate and [2-^13^C]pyruvate enables measurement of the flux through the pyruvate dehydrogenase (PDH) complex and TCA flux, respectively (116). A decrease in PDH flux, representative of reduced oxidative mitochondrial carbohydrate metabolism, was observed in the myocardium of DOX-treated rats for 3 weeks without impairment of cardiac function (117). After 6 weeks of treatment, the authors showed, in addition to reduced PDH activity, a decrease of TCA cycle flux and impaired cardiac function. This altered carbohydrate metabolism reflected the loss of mitochondrial integrity, which was not because of the oxidative stress in this study, and preceded cardiac function impairment.

The exploration of cardiac energetics in the clinic has been recently proposed. The authors found no difference in cardiac PCr/ATP ratio of anthracycline-treated women despite a 5% reduction in LVEF between the start and end treatment (118). This could be explained, at least in part, by the small number of patients in whom CMRS was possible (11 patients).

#### Nuclear Imaging

Several radiopharmaceuticals can be used as biomarkers of myocardial metabolism using nuclear imaging, the two best known being iodine-123 betamethyl-iodophenyl-pentadecanoic acid (BMIPP) for the assessment of myocardial FFA uptake and 2'-deoxy-2'-[^18^F]fluoro-D-glucose (FDG) for the assessment of cardiac glucose uptake. Because myocardial metabolism is tightly regulated, the heart switches from FFA metabolism to glycolysis in high-insulin/glucose levels and low oxygen by increasing its glucose transporter protein translocation to the plasma membrane (119). Hence, PET with FDG under fasting condition is preferred for oncology study (minimize myocardial uptake) but is performed under fasted condition or with glucose load after an overnight fasting for cardiac study (maximize myocardial uptake).

Early studies conducted two decades ago showed a significantly lower myocardial BMIPP uptake in patients treated with DOX (120) and taxanes (121), but other studies showed that only one in four (122), and one in six (123) patients displayed hypomyocardial BMIPP accumulation. Importantly, modeling of kinetics, which was measured by the acquisition of dynamic time sequences in the latter study, revealed a significant decrease in BMIPP flux in DOX-treated patients (123). This analysis more accurately reflects the features of fatty acid metabolism disorders by measuring the metabolic flux of the tracer rather than its accumulation in the myocardium. The lower cardiac uptake of BMIPP, which is a biomarker of impaired fatty acid beta-oxidation, was predictive of LV dysfunction (120).

An exciting exploration in cardio-oncology is ketone body imaging. This has been proposed with cardiac ^11^C-acetoacetate PET. As a ketone body, acetoacetate can be used as a substrate by the heart and be involved in cardioprotection through its antioxidant activity plus mitochondrial membrane repair (124, 125). Greater uptake and retention of ^11^C-acetoacetate in the myocardium was found in non-fasted rats treated for 6 weeks with DOX, which may be associated with mitochondrial membrane alteration (126). Although it has been studied only once in this field, ketone body imaging may hold promise as a theranostic approach.

In 2012, Borde et al. first described enhanced ^18^F-FDG uptake in the myocardium of DOX-treated patients, highlighting the ability of PET to early detect cardiotoxicity (127). Similar observations have been reproduced by others attempting to better understand the increased myocardial ^18^F-FDG uptake in animals and patients treated with chemotherapy. First, DOX dose-dependently increased myocardial metabolic flux of ^18^F-FDG measured by dynamic PET in the fasted mice (128). The same group demonstrated that a low pretreatment ^18^F-FDG standardized uptake value (SUV) in Hodgkin's disease patients may predict the development of chemotherapy-induced cardiotoxicity, which was subsequently detected by a higher myocardial ^18^F-FDG SUV (128). Another study showed that 12% of 121 patients with breast cancer treated with anthracycline or trastuzumab had increased ^18^F-FDG uptake in the right ventricle, which was significantly associated with cardiotoxicity (129). Second, increased LV ^18^F-FDG uptake correlated with LVEF decline after two cycles and at the end of DOX therapy in a retrospective study including a cohort of 43 patients (130). Another interesting study explored ^18^F-FDG myocardial uptake and myocardial perfusion (through ^99m^Tc-tetrofosmin SPECT) in a retrospective cohort of 332 patients followed for malignant disorders (131). As part of an oncologic PET protocol, patients were fasted to avoid myocardial ^18^F-FDG uptake: 36% of patients had no ^18^F-FDG uptake, 22.5% had diffuse ^18^F-FDG uptake, 8% had focal ^18^F-FDG uptake, and 30.5% had a focal uptake overlying the diffuse pattern ^18^F-FDG uptake. Among all the patients, multivariate logistic regression identified focal myocardial ^18^F-FDG uptake as a predictor of impaired LVEF and myocardial perfusion (131). It is important to bear in mind two interesting points. First, no direct mechanisms that could explain the increased cardiac ^18^F-FDG uptake have been explored in these reports. This could be because of the recruitment of inflammatory cells, switch to anaerobic glycolysis, or being associated with other pathological mechanisms. Second, the correlation between ^18^F-FDG uptake and LV function was made at the same time, which cannot directly prove the ability of early detection of cardiotoxicity before the decline of LV function. In terms of mechanisms and correlations, the increase in cardiac uptake of ^18^F-FDG seven days after DOX treatment in mice was directly correlated with oxidative stress and antioxidant mechanisms assessed by biochemical measurements (132). This is particularly interesting knowing the close relationship between metabolic imbalance (i.e., mismatch of oxidative metabolism plus reduced ATP production) and ROS generation in mitochondria (133, 134).

Chemotherapy-induced cardiotoxicity is not limited to an increase in ^18^F-FDG uptake. The SUV of ^18^F-FDG was significantly reduced in the fasted rats treated for 6 weeks (135) and in non-fasted rats treated for 4 weeks (136) with DOX. ^18^F-FDG PET could have detected a loss of cell viability and necrosis in these experimental models, which was associated with decreased LVEF (136). This supports the fact that dietary status is important in the cardiac ^18^F-FDG PET investigation.

With respect to antiangiogenic therapies, few reports have described the role of ^18^F-FDG PET. In 2011, a case report described decreased myocardial ^18^F-FDG uptake in patients treated with imatinib plus sorafenib who later developed a cardiac event (137). Later, O'Farrell et al. also showed an increase in ^18^F-FDG uptake 2–3 days after the introduction of sunitinib in mice and 5 days in rats (87). In another study, sunitnib induced higher ^18^F-FDG uptake after 1 week of treatment in fasted mice but not in non-fasted mice (138), highlighting once again a role of the dietary status on myocardial ^18^F-FDG uptake for further investigations. In both studies, this early side effect was associated with a switch from oxidative metabolism to glycolytic metabolism (138) and correlated with late myocardial hypertrophy measured after 6 weeks of treatment (139). Moreover, the metabolic flux of ^18^F-FDG from the blood to the cytoplasmatic glycolysis, measured by dynamic time sequence acquisition and kinetic modeling, was reduced after 3 weeks of treatment (87, 138) with sunitinib and was associated with an insulin resistance pattern (138).

### Mitochondrial Function Imaging

*In-vivo* assessment of cardiotoxicity-induced ROS production is tempting as there is a close relationship between altered circulation, metabolism, and oxidative stress. ^18^F-labeled analog of dihydroethidium (^18^F-DHMT) is a radioactive compound that can assess free radicals because it is trapped in the cell when oxidized by ROS (140, 141). In an initial *in-vivo* study in mice, the authors reported a 2-fold increase in cardiac retention of ^18^F-DHMT after a single injection of DOX, which revealed ROS production compared with controls (141). This observation was later confirmed with an increased cardiac uptake of ^18^F-DHMT in DOX-treated rats following 4 and 6 weeks of treatment (142). Interestingly, no impairment of cardiac function was found after 4 weeks of treatment, but 6 weeks of DOX treatment induced a decrease in LVEF (142). In another study, dynamic time sequence ^18^F-DHMT PET and kinetic modeling confirmed higher absolute quantification of myocardial ROS production in beagle dogs following 2 weeks of DOX treatment (143).

Similarly, new radiopharmaceuticals have been developed to assess early DOX myocardial cardiotoxicity detection, such as ^18^F-labeled lipophilic cation PET tracers (144). Its principle is to image mitochondrial damage by ^18^F-labeled lipophilic tracers, which diffuse across mitochondrial membranes depending upon the mitochondrial membrane potential (144). The tracers will therefore accumulate in cardiac tissue in case of mitochondrial damage, which is one of the possible mechanisms of myocardial cardiotoxicity of DOX, allowing early detection of its cardiotoxicity.

In SPECT imaging, in the same perspective, the usual ^99m^Tc- sestamibi, which is used to assess myocardial perfusion, is also a lipophilic cation and so its myocardial distribution depends on the mitochondrial membrane potential additionally to regional myocardial perfusion. Safee et al. recently demonstrated in a rat model a correction tool to free the ^99m^Tc-sestamibi from its perfusion imaging, to assess only the mitochondrial potential, and thus, its possible perturbation by anthracyclines (145). They proposed to correct the ^99m^Tc-sestamibi with a lipophilic uncharged radiotracer that would thus be a perfusion tracer independent of the mitochondrial membrane potential [the bis (N-ethoxy-N-ethyldithiocarbamato)nitrido ^99m^Tc(V)]. The latter ^99m^Tc-NOET would, therefore, be able to detect DOX cardiotoxicity through its mitochondrial damage.

## Perspectives

We are convinced that the assessment of the mechanisms of anticancer drug cardiotoxicity by imaging is a cornerstone in the new era of cardio-oncology. Table 2 supports our assertion by summarizing studies that demonstrate DOX-induced cardiotoxicity early before overt LVEF impairment (Table 2).

**Table 2:** This table summarizes early perfusion, metabolic and mitochondrial function imaging findings suggestive of DOX myocardial toxicity that subsequently revealed impaired left ventricle ejection fraction.

**Reference**	**Early myocardial toxicity with no overt cardiac dysfunction**	**Late cardiac dysfunction**	**Species**
Saito et al. (120)	Reduced ^123^I-BMIPP [2 to 3 weeks]	Decreased LVEF [variable]	Human
Maslov et al. (110)	Decreased PCr/ATP ratio [6 weeks]	Systolic and diastolic dysfunction [8 and 10 weeks]	Mouse
Bauckneht et al. (128)	Lower pre-treatment ^18^F-FDG Increased ^18^F-FDG [4-6 weeks and 6 months follow up]	Decreased LVEF [median = 27 months, range 8-96]	Human
Boutagy et al. (142)	Increased ^18^F-DHMT [4 weeks]	Decreased LVEF [6 weeks]	Rat
Timm et al. (117)	Decreased PDH flux [3 weeks]	Decreased LVEF [6 weeks]	Rat
Galán-Arriola et al. (31)	Decreased CMR-determined myocardial perfusion Decreased CFR [weeks 6]	Decreased LVEF [weeks 16]	Pig

*[time] = from the beginning of treatment to the assessment of alteration on imaging*.

*CFR, coronary flow reserve; CMR, cardiac MRI; ^18^F-DHMT, ^18^F-labeled analog of dihydroethidium; DOX, doxorubicin; ^18^F-FDG, ^18^F-Fluoro-D-glucose; ^123^I-BMIPP, ^123^I-Betamethyl-iodophenyl-pentadecanoic acid; LVEF, left ventricle ejection fraction; PCr, phosphocreatine; PDH, pyruvate dehydrogenase*.

### Imaging Opportunities

We have seen throughout this review that most studies have been conducted in animal models. We are confident that this research has been and will be of great importance for the development of a standardized protocol to predict drug-related cardiotoxicity and to test preventive interventions.

Early detection of metabolism and vascular alteration is paramount to prevent DOX-induced permanent cardiac dysfunction (Table 2) and could be extended to other anticancer drugs since several vascular and metabolic cardiotoxic effects have been described in this review (Table 1). The assessment of myocardial cardiotoxicity by CMR seems to be of interest, to seek other complications of oncologic therapies such as ICI-induced cardiotoxicity. The major cardiotoxicity reported in this therapeutic class is myocarditis, with CMR being of great importance when suspected (146). Although not a commonly used modality for myocardial inflammation (147), increased ^18^F-FDG uptake on PET could be found in myocarditis, including in ICI myocarditis (148). Interestingly, ^18^F-FDG uptake has also been reported as a marker of anthracyclines cardiotoxicity, either *via* inflammatory response or altered myocardial metabolism (149). Fusion between ^18^F-FDG and CMR have also been reported (148) for simultaneous vascular, metabolic, and functional imaging and may benefit from creatine measurement with proton CMRS (150) since creatine is decreased in both ischemic (151) and non-ischemic (152) cardiovascular disease.

### Clinical Feasibilities

Because most studies of perfusion and metabolic imaging have been performed in animal models, their clinical relevance in routine practice is questionable. Anyhow, further clinical studies are required to ensure the utility of early detection of anticancer drugs.

Cardiac magnetic resonance imaging appears to be a non-invasive, radiation-free tool for monitoring patients with cancer, capable of imaging microcirculation, metabolism, and myocardial inflammation, which could be offered routinely before and after the introduction of an anticancer drug. We believe that CMR could be a justifiable perfusion approach as a part of standard patient care. Indeed, we have seen that altered myocardial perfusion in large animal models has been reported by resting myocardial perfusion on CMR (31). Multiple other CMR parameters have been reported to be related to cardiotoxicity of anticancer drugs (153–156), so the addition of a rapid perfusion sequence to the CMR protocol would be sufficient to obtain an argument for cardiotoxic effect. As the gold standard, CMR would also provide an accurate evaluation of LVEF. Unfortunately, LVEF assessment is so far performed in daily practice by echocardiography because of the lack of access to CMR. This would be the only limitation we see for its routine integration into the health care of patients with cancer.

We believe that the use of nuclear perfusion imaging in daily practice is difficult to justify. One of the main possible obstacles is the use of radiation and the cost of the technique that would allow assessment of myocardial perfusion without assessing oncologic follow-up. Nevertheless, it may be interesting to consider the integration of ^18^F-FDG PET in the follow-up of patients with cancer in order to assess tumor progression and, at the same time, to look for possible cardiotoxic effects. Indeed, the most PET scans for oncology monitoring use ^18^F-FDG, which is also, as mentioned earlier, sensitive to myocardial metabolic imbalance and also to myocardial inflammation. This capability of PET for whole-body imaging would be attractive in patients with cancer to concomitantly allow imaging of tumor progression in addition to an assessment of myocardial toxicity, thus providing a unique modality. We believe that further studies regarding the place of PET imaging in the future of cardio-oncology are required.

## Conclusion

Early detection of cardiotoxicity is crucial and offers the opportunity for early therapeutic intervention. In this review, we have shown that perfusion imaging, metabolic imaging, and mitochondrial function imaging are capable of assessing myocardial cardiotoxic effects of cancer therapeutics before irreversible cardiac damage occurs (Figure 1, Table 2). Knowledge of these possible early imaging findings in anticancer drug-related myocardial toxicity could change the paradigm of “late-onset cardiotoxicity.” Earlier detection would allow for better prevention, with specific therapeutics attempting in part to reduce oxidative stress. Current guidelines on cardiotoxicity do not include myocardial and metabolic perfusion imaging, but in light of this review, it may be worthwhile to add these parameters to better detect and prevent dramatic progression.

## Author Contributions

FC and JS contributed equally to this study and wrote the manuscript. FT did proofreading and provided useful advice. All authors contributed to the article and approved the submitted version.

## Funding

This study was performed by a laboratory member of the France Life Imaging Network (grant ANR-11-INBS-0006).

## Conflict of Interest

The authors declare that the research was conducted in the absence of any commercial or financial relationships that could be construed as a potential conflict of interest.

## Publisher's Note

All claims expressed in this article are solely those of the authors and do not necessarily represent those of their affiliated organizations, or those of the publisher, the editors and the reviewers. Any product that may be evaluated in this article, or claim that may be made by its manufacturer, is not guaranteed or endorsed by the publisher.
